# Lactylation-related gene signature accurately predicts prognosis and immunotherapy response in gastric cancer

**DOI:** 10.3389/fonc.2024.1485580

**Published:** 2024-11-28

**Authors:** Xuezeng Sun, Haifeng Dong, Rishun Su, Jingyao Chen, Wenchao Li, Songcheng Yin, Changhua Zhang

**Affiliations:** ^1^ Guangdong Provincial Key Laboratory of Digestive Cancer Research, The Seventh Affiliated Hospital of Sun Yat-sen University, Shenzhen, Guangdong, China; ^2^ Hospital for Skin Diseases, Shandong First Medical University, Jinan, Shandong, China

**Keywords:** gastric cancer, lactylation-related genes, prognostic signature, PD-L1, immunotherapy

## Abstract

**Background:**

Gastric cancer (GC) is a malignant tumor associated with significant rates of morbidity and mortality. Hence, developing efficient predictive models and directing clinical interventions in GC is crucial. Lactylation of proteins is detected in gastric cancer tumors and is linked to the advancement of gastric cancer.

**Methods:**

The The Cancer Genome Atlas (TCGA) was utilized to analyze the gene expression levels associated with lactylation. A genetic pattern linked to lactylation was created using Univariate Cox regression and least absolute shrinkage and selection operator (LASSO) regression. The predictive ability of the model was evaluated and confirmed in the Gene Expression Omnibus (GEO) cohort, where patients were divided into two risk groups based on their scores. The study examined the relationship between gene expression and the presence of immune cells in the context of immunotherapy treatment. *In vitro* cytotoxicity assays, ELISA and PD-1 and PD-L1interaction assays were used to assess the expression of PD-L1 while knocking down SLC16A7.

**Results:**

29 predictive lactylation-related genes with differential expression were discovered. A signature consisting of three genes was developed and confirmed. Patients who had higher risk scores experienced worse clinical results. The group with lower risk showed increased Tumor Immune Dysfunction and Exclusion (TIDE) score and greater responsiveness to immunotherapy. The tumor tissues secrete more lactate acid than normal tissues and express more PD-L1 than normal tissues, that is, lactate acid promotes the immune evasion of tumor cells. In GC, the lactylation-related signature showed strong predictive accuracy. Utilizing both anti-lactylation and anti-PD-L1 may prove to be an effective approach for treating GC in clinical settings. We further proved that one of the lactate metabolism related genes, SCL16A7 could promote the expression of PD-L1 in GC cells.

**Conclusion:**

The risk model not only provides a basis for better prognosis in GC patients, but also is a potential prognostic indicator to distinguish the molecular and immune characteristics, and the response from Immune checkpoint inhibitors (ICI) therapy and chemotherapy in GC.

## 1 Introduction

Gastric cancer (GC), a prevalent malignant tumor globally, poses a significant threat to human health (1). GC arises from a complex interplay of genetic predisposition, dietary habits, Helicobacter pylori infection, and environmental factors, progressing through different stages and involving a variety of influences. Most cases of early-stage GC are often diagnosed late because of its unusual symptoms. Patients with GC usually have a dismal prognosis with a significant chance of distant metastasis and local recurrence (2–4). Advancements in GC research have revealed that cellular metabolic inefficiency plays a crucial role in the progression of GC, indicating that GC is not exclusively attributed to particular gene abnormalities (5–8). Growing evidence indicates that tumor metabolism affects immune cells by releasing metabolites such as lactate and arginine, which are essential for the development and progression of cancer. This metabolic competition between immune cells and tumors leads to nutrient deprivation (9).

Tumors arise and progress due to a variety of factors, leading to diverse gene expression, cell morphology, and metabolic features. This heterogeneity can be attributed to factors such as gene mutations, the tumor microenvironment (TME), and changes in homeostasis (10). Though the tumor microenvironment usually presents many different characteristics than normal tissues, but the tumor cell growth also need adenosine triphosphate (ATP) as energy support. According to the Warburg effect, even if there is sufficient oxygen supply, tumor cells still choose glycolysis to obtain energy. Excess lactic acid further secreted outside the cell makes the tumor microenvironment become conducive to the growth of tumors, affecting tumor cell proliferation, immune escape and other behaviors. New studies indicate that lactic acid is essential in the tumor microenvironment and is closely associated with tumor cells’ capacity to avoid detection by the immune system. Its key roles include controlling immune cell metabolism, suppressing the activation and growth of various immune cells, and serving as a signaling molecule to regulate the immune response of tumor cells, affecting immune surveillance and evasion mechanisms (11–13).

**Figure 1:**
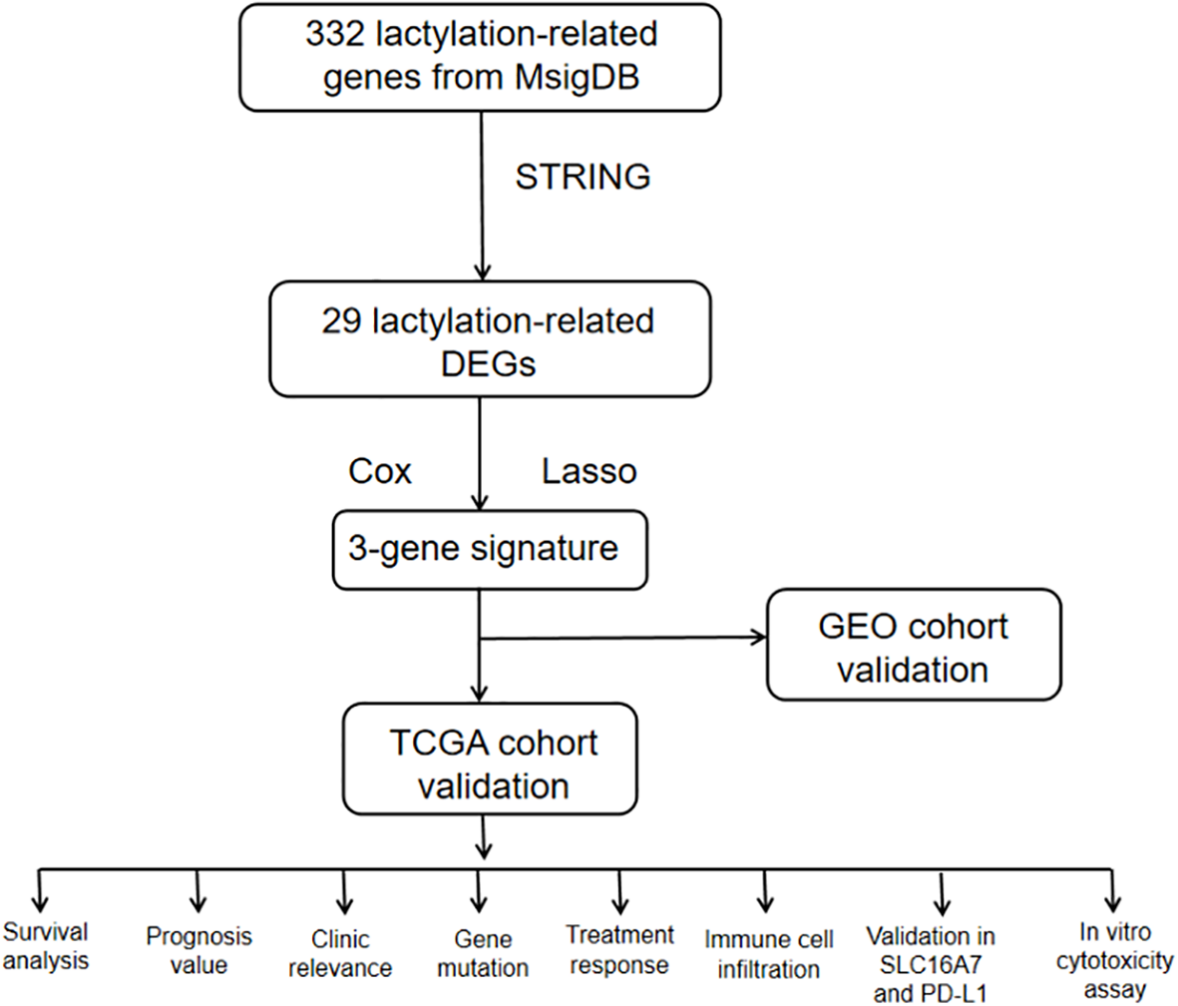
The workflow of study.

By examining RNA-seq data and clinical information from The Cancer Genome Atlas (TCGA) and Gene Expression Omnibus (GEO) database, we identified genes linked to lactatylation that exhibited varying expression in gastric cancer tissues compared to normal tissues. This information enabled us to create a predictive marker for genes associated with lactatylation. Following this, a study was researched to explore the relationship between lactatylation-associated genes and immune-infiltrating cells within the tumor microenvironment, along with the effects of immunotherapy. Furthermore, experiments were conducted to confirm the findings obtained through bioinformatics analysis. The study’s flowchart is depicted in **Figure 1**.

## 2 Methods

### 2.1 Data acquisition and processing

Access to the Molecular Signatures database v7.4 (MSigDB) is available through the use of the search terms ‘lactate’ (14) at https://www.gsea-msigdb.org/gsea/msigdb. Eight pathways involving lactate were identified: GOBP LACTATE TRANSMEMBRANE TRANSPORT, GOMF LACTATE DEHYDROGENASE ACTIVITY, HP ABNORMAL BRAIN LACTATE LEVEL BY MRS, HP ABNORMAL LACTATE DEHYDROGENASE LEVEL, HP ELEVATED LACTATE PYRUVATE RATIO, HP INCREASED CIRCULATING LACTATE DEHYDROGENASE CONCENTRATION, HP INCREASED CSF LACTATE, and HP INCREASED SERUM LACTATE. After eliminating any redundant genes, a total of 322 genes related to lactatylation were combined. The “limma” package in R was used to examine the mRNA expression matrix and genes linked to lactatylation, then the expression matrix was extracted specifically for lactatylation-related genes. Among these, 29 genes were recognized as genes associated with lactylation that showed varying expression levels between tumor samples and neighboring tissues, exhibiting a fold change (FC) exceeding 1.5 and a false discovery rate (FDR) below 0.05.

### 2.2 Identification of lactylation−related genes in gastric cancer

We used “STRING” and the ‘corrplot’ package to compute Pearson correlations at the transcriptional level and explore the co-expression patterns of important genes.

### 2.3 Construction and validation of prognostic risk models

P<0.05 was chosen as the cutoff for prognostic gene screening. The risk model was then further compressed using a Lasso regression analysis to minimize the number of genes. By building a penalty function to compress the number of coefficients and set some coefficients to zero, the Lasso technique is a compressed estimation that results in a more refined model. As a result, it still has the benefit of subset contraction and permits biased estimation for data with complicated collinearity. The Lasso model is more effective at resolving the multicollinearity issue in regression analysis and may realize the choice of variables when estimating parameters. The risk score was calculated as follows: Risk score = ∑multivariate Cox regression coefficient x gene expression value. Patients were divided into high-risk and low-risk categories based on the median risk score. The TCGA and GEO datasets were designated as the training and testing sets, respectively, for the prediction of overall survival using Kaplan-Meier survival analysis. Furthermore, genes linked to lactylation were subjected to survival analysis using the Kaplan-Meier technique. Next, the risk score and clinical characteristics were combined for both univariate and multivariate analyses. Age, gender, the TNM stage, grade, and the risk score were utilized as covariates. ROC analysis was utilized to assess the precision of the risk score signature in predicting survival rates at 1, 3, and 5 years. Specificity and sensitivity were evaluated by calculating the areas under the curves (AUC) with the ‘survival ROC’ R package. After being found to be lactylation-related genes, the genes were applied to the prediction of GC prognosis.

### 2.4 Construction of nomogram and clinical correlation analysis

TCGA cohort was considered as training group and GEO cohort was deemed as test group, then we combined them together to further analyze. Age, gender, grade, stage, survival time, and survival status were utilized alongside the risk score from TCGA-STAD for univariate and multivariate Cox proportional hazard regression analyses, investigating the influence of lactylation-related gene signature on overall survival (15). A nomogram was created to predict the 1-year, 3-year, and 5-year overall survival rates in TCGA-STAD using the calibration functions provided by the ‘rms’ software. The nomogram scoring method assigns a numeric score to each variable, and the total score for each instance is determined by adding up the scores of all variables. The accuracy of the 1-, 3-, and 5-year models was evaluated using the Kaplan-Meier method, and calibration curves were created to compare the predicted and actual OS rate.The dependability of the model was evaluated through decision curve analysis (DCA) (16). Afterward, a study was done to analyze how risk score is related to clinical factors, and then a heatmap and circle graph were generated.

### 2.5 Analysis of tumor immune signatures and function enrichment for lactylation risk

The research included examining connections by using the ssGSEA method to explore the relationship between the lactylation score and immune cells.GSEA website (https://www.gsea-msigdb.org) provided the Kyoto Encyclopedia of Genes and Genomes (KEGG) pathway files. The enhanced functional pathways in gastric cancer subtypes were displayed as a heatmap utilizing the ‘GSEABase’ and ‘GSVA’ packages in R. Furthermore, the mutation information was evaluated and summarized with the ‘maftools’ package in R.

### 2.6 Analysis of immunotherapy for immune subtypes

The study focused on Tumor Mutational Burden (TMB), which refers to the number of mutations in the coding sequence area of the longest transcript sequence, normalized per million bases. TMB encompasses point mutations and indels. Higher TMB can lead to the production of more neoantigens, increasing the likelihood of T cell recognition and enhancing the effectiveness of immunotherapy. The use of immune checkpoint inhibitors (ICIs) in the treatment of microsatellite instability (MSI) has been associated with improvements in results. MSI is caused by changes in the length of microsatellite (MS) sequences, which result from mutations during DNA replication. This condition is caused by defects in mismatch repair (MMR). The researchers utilized the TIDE (Tumor Immune Dysfunction and Exclusion) analysis available on the TIDE online database to predict the efficacy of immune checkpoint inhibitors (ICIs) in treatment. Through this analysis, researchers obtained TIDE prediction scores for each sample, which indicated the likelihood of immune evasion and the potential response to ICIs treatment. Additionally, the study assessed TIDE, MSI, T-cell dysfunction, and exclusion scores for each TCGA-STAD sample using the TIDE online tool. This evaluation aimed to assess the potential benefits of immunotherapy across various lactylation score groups.

### 2.7 Cell culture and chemicals

SNU-719 and AGS gastric cancer cell lines were cultured in DMEM medium (KeyGEN, China) supplemented with 10% fetal bovine serum (BI, Israel). The incubator was maintained at a temperature of 37 degrees Celsius and a carbon dioxide concentration of 5%.To create a 5 mM stock solution, syrosingopine, a suppressor of MCTs, was kept at -20°C in a DMSO solution. Following this, the cells were treated with varying levels of syrosingopine for a duration of 48 hours. The sodium L-lactate was purchased from Sigma-Aldrich.

### 2.8 Real-time polymerase chain reaction

RNA was extracted from 10 samples of both healthy and gastric cancer tissues using TRIzol reagent (Invitrogen, Thermo Fisher Scientific, Inc.) at Seventh Hospital of Sun Yat-Sen University from 2022 to 2023. The reverse transcription reaction utilized the PrimeScript ™ RT reagent kit by TaKaRa. GAPDH was used to normalize the mRNA levels of CD274, COL4A1, SLC16A7, and IRAK1.The primer sequences for the 3 genes, CD274 and GAPDH are listed in **Supplementary Table S3**. Normalized CT values were used to calculate fold variances within each group.

### 2.9 Westren blot

Proteins were extracted from 5 paired normal and gastric cancer samples collected between 2022 and 2023 at Seventh Hospital of Sun Yat-Sen University in Guangdong, China by lysis buffer from KeyGEN. Lysates were then separated by Sodium Dodecyl Sulfate-Polyacrylamide Gel Electrophoresis (SDS-PAGE) and transferred onto a polyvinylidene fluoride (PVDF) membrane. After blocking, the membrane was incubated with a primary antibody. Following two rinses with tris-buffered saline (TBS), the membrane was treated with a horseradish peroxidase-conjugated secondary antibody, washed, and visualized using an enhanced chemiluminescence (ECL) detection system and a LAS-4000 (GE Healthcare). The relative concentrations were measured using Bradford from KeyGEN. Equal amounts of protein with matching concentration and volume were added. PTM Bio supplied the Anti-l-Lactyl Lysine Rabbit mAb, while Fude Biology provided the Antibody targeting β-actin, PD-L1, and Goat anti-Mouse/Rabbit IgG.

### 2.10 Immunohistochemistry

To detect the expression of SLC16A7, IHC was performed. The process included removing paraffin and hydrating tissue sections embedded in paraffin by using xylene and various concentrations of ethanol. To recover the antigen, the sections were boiled in a citrate buffer solution with a pH of 6.0 for 10 minutes. To block endogenous peroxidase activity, the slides were treated with 3% hydrogen peroxide for 10 minutes. Afterward, the sections were treated with 5% goat serum and blocked for 30 minutes at room temperature. In this research, the main antibody employed was the Anti-SLC16A7 Polyclonal Antibody (K008867P) from Solarbio, which was diluted at a ratio of 1:100. The sections were then left to incubate with the antibody overnight at a temperature of 4°C. The following day, the segments were rinsed with PBS and then treated with the suitable secondary antibody, goat anti-rabbit IgG H-conjugated.

### 2.11 Extraction and cultivation of T cells

Peripheral blood mononuclear cells (PBMCs) were isolated from whole blood buffy coats using Ficoll 400 gradient centrifugation. The extracted PBMCs were stimulated for 48 hours in 12-well plates (approximately 5×10^6 cells per well) pre-coated with T cell activators (anti-CD3 and anti-CD28 from STEMCELL Technologies, 10971) in a serum-free medium specifically designed for lymphocytes (SuperCulture, L500), supplemented with 0.5‰ IL-2. The stimulation process lasted 5-7 days, after which T cells were obtained.

### 2.12 PD- L1 and PD-1 interaction assays

GC cells were incubated with recombinant human PD-1 Fc protein (R&D Systems) for 1 hour. Subsequently, anti-human Alexa Fluor 488 dye-conjugated secondary antibodies (Life Technologies) were applied for 1 hour. The cells were then examined using a confocal laser-scanning microscope (Carl Zeiss).

### 2.13 *In vitro* cytotoxicity assay

SNU-719 and AGS cells were cultured in 96-well plates until they reached approximately 70% confluency. To investigate the cytotoxic effect of T cells on tumor cells, GFP-expressing tumor cells were co-cultured with activated primary human T cells (derived from PBMCs activated by anti-CD3/CD28 and IL-2) for 24 hours. IncuCyte™ Cytotox Reagents were added to the culture (tumor cells/T cells at a 5:1 ratio) to assess cytotoxicity. Cytotox Red Reagent was introduced at the 24-hour mark to distinguish dead cells. Importantly, these reagents do not interfere with the growth of normal cells. When cells undergo apoptosis or necrosis, the loss of plasma membrane integrity allows the reagent to bind to DNA, resulting in enhanced red fluorescence. Cells were then analyzed using a confocal laser scanning microscope, where cells displaying red fluorescence were classified as dead.

In a parallel experiment, tumor cells and T cells were co-cultured in six-well plates under the same conditions for 24 hours. After co-culture, T cells were carefully washed away using PBS, and the remaining adherent tumor cells were fixed with 4% paraformaldehyde for 15 minutes. Following fixation, the tumor cells were stained with crystal violet for 30 minutes to visualize and quantify the remaining viable cells.

### 2.14 Enzyme-linked immunosorbent assay

The supernatant from the co-cultured cells was collected and analyzed to measure the levels of interferon-gamma (IFN-γ), tumor necrosis factor alpha (TNF-α), and granzyme B (GmzB) using ELISA kits (MEIMIAN). The supernatant was first centrifuged at 4000 rpm for 20 minutes at 4°C to remove any debris, and the cleared supernatants were then stored at -80°C until they were ready for the ELISA assay. The ELISA kits were used following the manufacturer’s instructions to ensure accurate measurements of the cytokine and enzyme levels in the supernatant.

### 2.15 Immunofluorescence staining

Collect the supernatants from the gastric cancer cell cultures of the control group and the SLC16A7 knockdown group after 48 hours and add them to the M0 macrophages. Continue to culture for another 48 hours. Cells subjected to different treatments were fixed with 4% paraformaldehyde and permeabilized using 0.1% Triton X-100. Following a 1% BSA blocking step, the cells were incubated overnight at 4°C with CD163 (Proteintech) antibody. Afterward, fluorescein-labeled secondary antibodies were added and incubated for 1 hour at room temperature, followed by DAPI staining to visualize the nuclei. Images were captured using a laser confocal microscope (LEICA DMi8).

### 2.16 Statistical analysis

Statistical analyses were conducted using R software 4.2.2 or GraphPad 8, with a p-value < 0.05 considered statistically significant unless stated otherwise. Ns, *, **, ***, and **** stand for p-value >0.05, p-value <=0.05, p value <=0.01, p value <=0.001, and p value <=0.0001, separately. The analysis of survival was conducted utilizing the R software packages ‘survival’ and ‘survminer’. The Wilcoxon test was utilized for comparing two groups, while the Kruskal-Wallis test was used for comparing more than two groups.

## 3 Results

### 3.1 Identification of lactylation−related genes in gastric cancer

**Figure 2:**
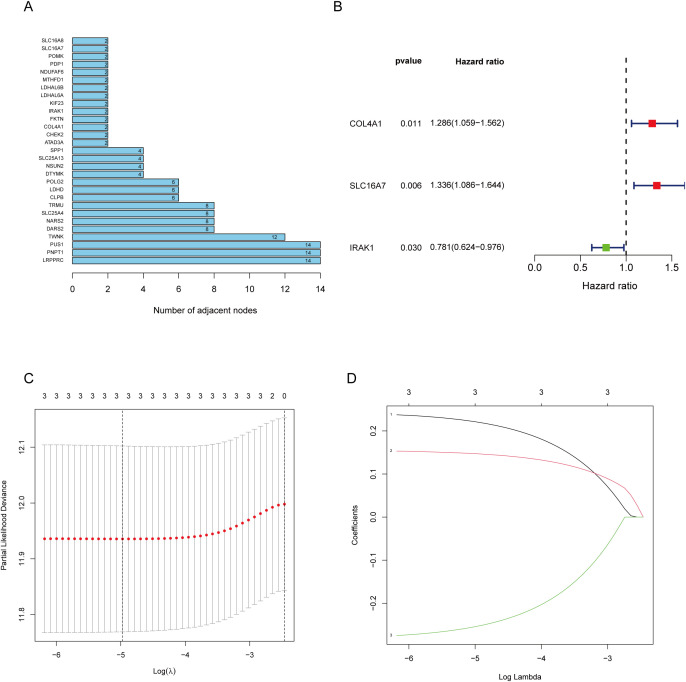
Screening of genes via STRING and filtrating lactylation-related genes. **(A)** 229 genes selected by STRING and their relationships. **(B)** Univariate Cox regression analysis to screen 29 prognosis-related genes. **(C, D)** LASSO coefficient curves of prognosis-related genes.

The workflow of the lactate-related gene signature analysis is demonstrated in **Figure 1**. Through the analysis of the MSigDB database, researchers have identified eight lactylation-related pathways and 322 genes that exhibit significant upregulation in GC tissues (**Supplementary Tables S1, 2**). This discovery suggests a potential direct association between these pathways and the development and progression of GC. To further explore the relationship between these genes, the researchers utilized the STRING database for protein interaction analysis. The results revealed a comprehensive and robust network of interactions among the 29 key genes, indicating potential functional associations and cooperation among them. This information could provide valuable insights into the underlying mechanisms and potential therapeutic targets related to lactylation and GC. (**Figure 2A**).

### 3.2 Development and verification of predictive risk models

The 29 key genes were applied into the unvariate cox regression (**Figure 2B**) and LASSO algorithm (**Figures 2C, D**), yielding 3 lactylation-related genes (COL4A1, SLC16A7, and IRAK1) (**Table 1**). Risk score = (0.22*COL4A1) + (0.14* SLC16A7) - (0.25*IRAK1).

**Table 1:** The coefficients of three genes in the risk model formula.

Lactylation-related genes	coefficients
**COL4A1**	0.220712497208269
**SLC16A7**	0.147004007788101
**IRAK1**	-0.253487009805723

**Figure 3:**
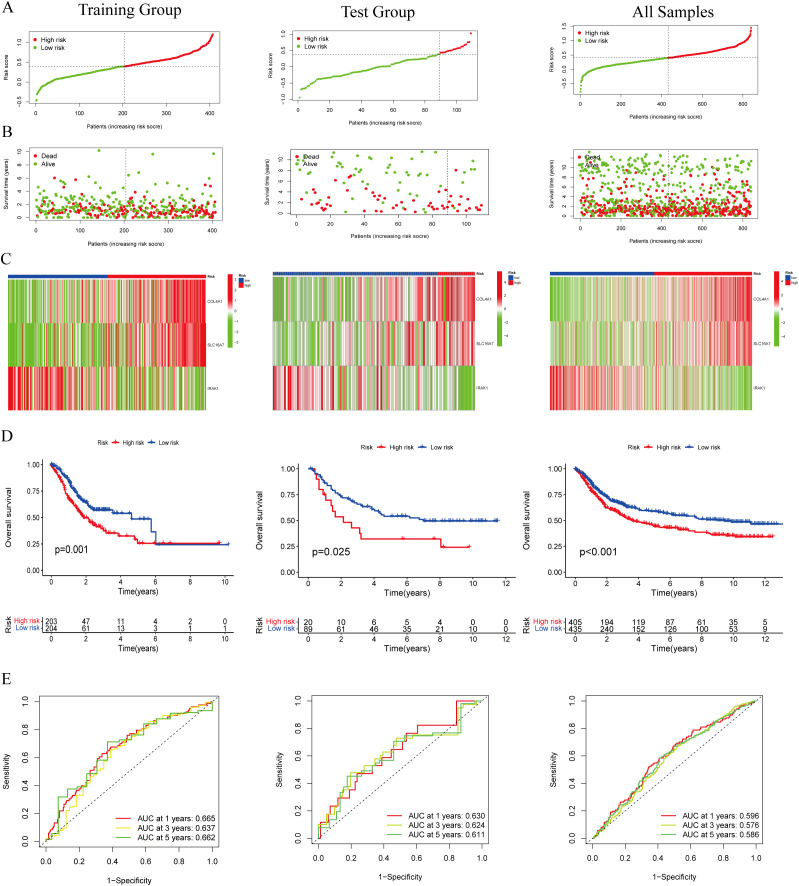
Evaluation of the lactylation-related genes signature in the TCGA-STAD and GEO cohort. **(A, B)** The distribution of the risk scores and scatter plots of survival in patients in the training group, text group, and all samples. **(C)** Prognostic signature signal heatmaps in the different group **(D)** The Kaplan-Meter curve analysis of the low-and –high-risk groups in the different group. **(E)** Receiver operating characteristics (ROC) curve analysis of the signature in the different group.

A total of 403 individuals diagnosed with GC in the TCGA group and 109 individuals diagnosed with GC in the GEO group were categorized into high-risk and low-risk categories based on their median risk scores. To determine the risk score for each patient, a calculation was performed, which then allowed for their classification into either the high-risk or low-risk group. This categorization based on risk scores helped in assessing the prognosis and potential outcomes for patients in these different risk categories (**Figure 3A**). The high-risk category showed an increased number of fatalities (**Figure 3B**), while the heatmap illustrated the spread of these 3 genes across various risk categories (**Figure 3C**). We employed survival curves to assess the predictive significance of the model on overall survival (OS) in GC. The results showed that the high-risk group had significantly lower OS rates compared to the low-risk group in both the training and test groups. This trend was also observed when considering the overall samples, indicating that the risk score model had a meaningful predictive value for OS in GC (**Figure 3D**). ROC analysis was performed to assess the predictive precision of the model. The AUC values for the training group, test group, and overall samples over a 5-year period were 0.662, 0.611, and 0.586, as shown in **Figure 3E**. The findings indicate the lactylation-related genes signature serves as a valuable predictive model for GC. Except for IRAK1, high expressions of COL4A1 and SLC16A7 predicted a poor prognosis (**Supplementary Figure S1**).

**Figure 4:**
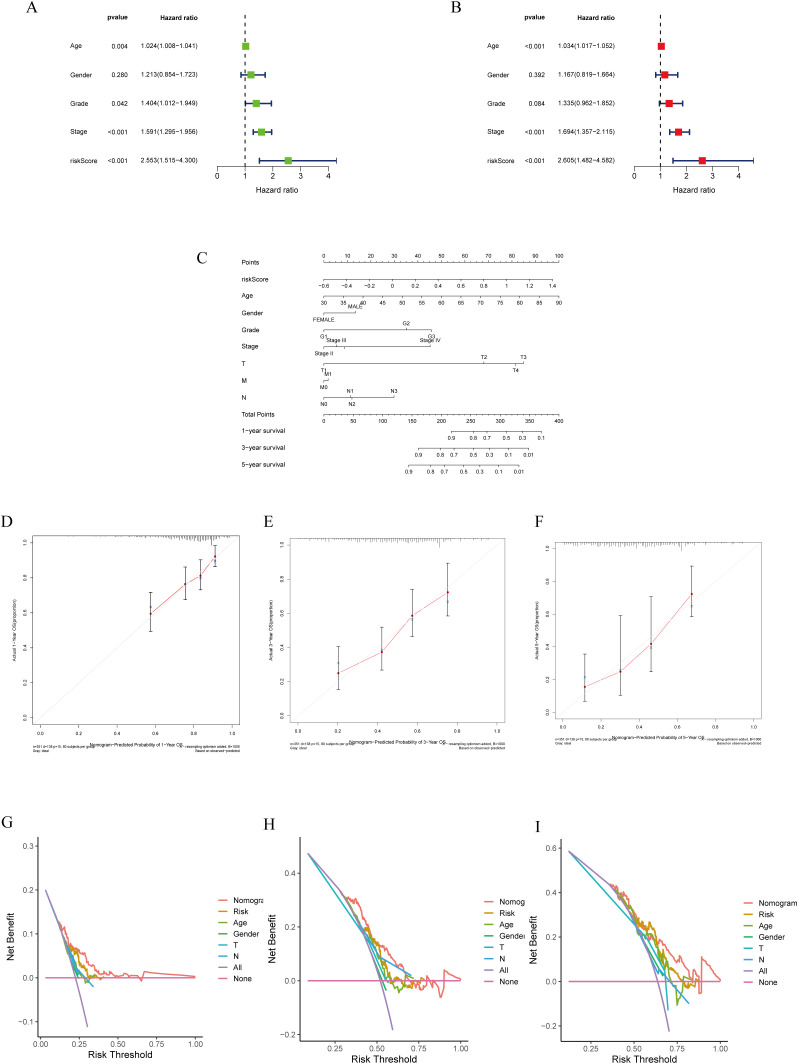
Construction of a nomogram model integrated with the risk score. **(A, B)** Univariate and multivariate Cox analyses included different clinicopathologic features. **(C)** Nomogram model for predicting the 1-, 3-, and 5 –year OS of GC patients. **(D–F)** The calibration plots for 1-, 3- and 5 years in the TCGA-STAD. **(G–I)** Decision curve for nomogram 1-, 3- and 5- years in the TCGA-STAD.

Cox regression analysis was employed to evaluate the significance of the prognostic models generated using lactylation-related genes as risk factors for GC. Univariate Cox regression analysis was conducted to assess the impact of various factors on patient prognosis. The results indicated that factors such as age, tumor stage, grade, and risk score significantly influenced patient prognosis (**Figure 4A**). Multivariate COX regression analysis revealed that the risk model was an autonomous predictor for GC outcomes (P<0.001, HR = 2.605, 95% CI = 1.482-4.582) as depicted in **Figure 4B**. A nomogram was developed using clinicopathological parameters to evaluate the effectiveness of risk models in clinical applications. The researchers conducted risk assessments to predict survival rates at 1-, 3-, and 5-year intervals (**Figure 4C**). The calibration graph demonstrated a strong agreement between the projected survival rates and the observed survival rates after 1-, 3-, and 5-years (**Figures 4D–F**). Additionally, according to the DCA, genes related to lactylation were found to be more beneficial in predicting the 1-, 3-, and 5-year overall survival rates compared to factors such as stage, age, and gender (**Figures 4G–I**).

### 3.3 Analysis of immune cell infiltration, functional enrichment, and gene mutations

**Figure 5:**
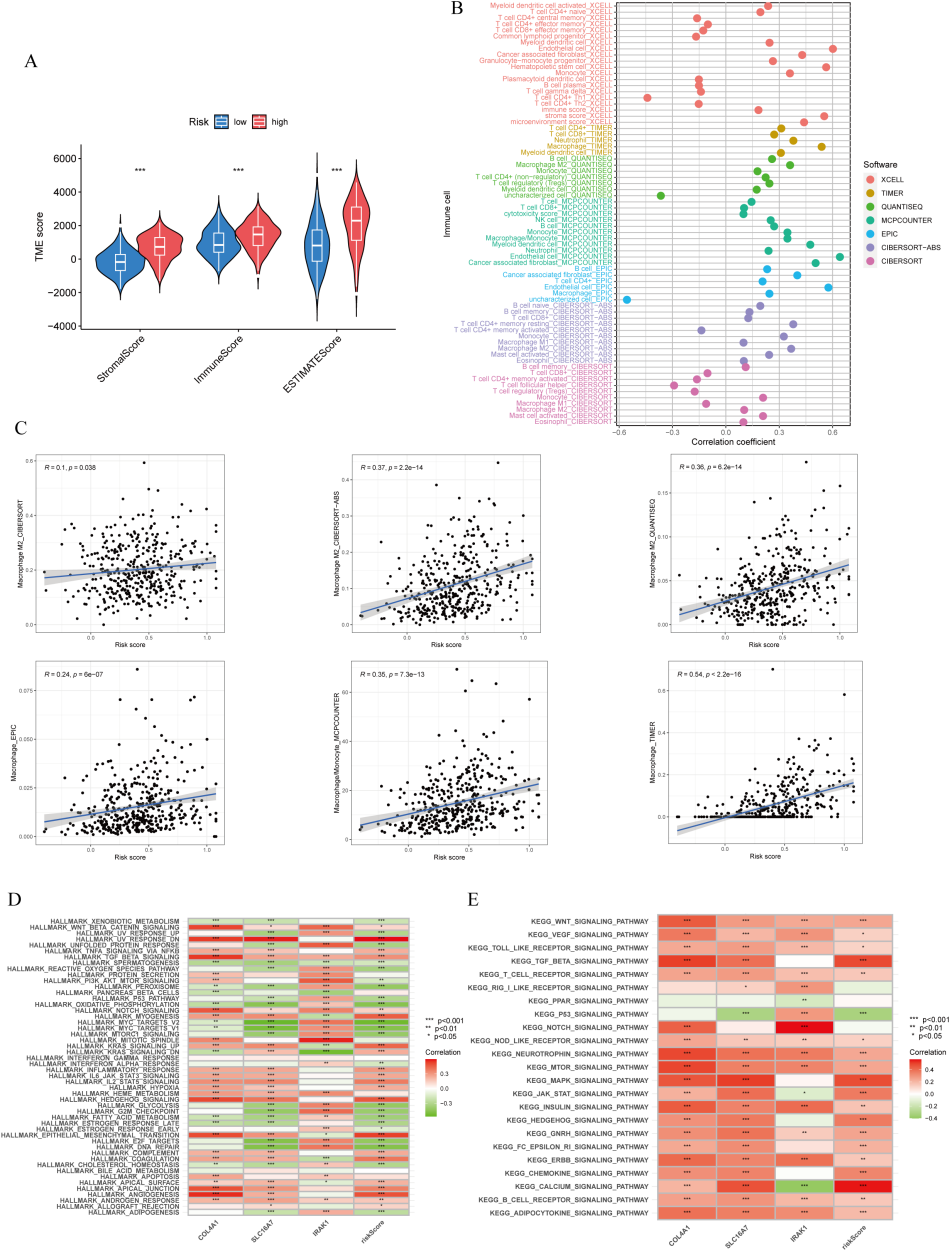
Immune cells infiltration and function enrichment analysis. **(A)** Correlation between lactylationscore and the tumor microenvironment of gastric cancer assessed using the ESTIMATE algorithm. **(B, C)** The correlation between lactylation score and immune cell infiltration by various immunocytes analysis methods. **(D, E)** GSVA analysis of lactylation score and lactylation-related genes. *p<0.05; **p<0.01;***p<0.001.

The ESTIMATE algorithm analysis revealed that the high lactylation score group had significantly elevated stromal and immune scores compared to the low lactylation score group. This suggests that the high lactylation score group had a higher proportion of stromal and immune cells infiltrating the tumor. Furthermore, the ESTIMATE score was significantly higher in the high lactylation score group, indicating an inverse association between lactylation score and tumor purity. In other words, higher lactylation scores were associated with lower tumor purity. (**Figure 5A**). By examining different immunocyte analysis methods, we conducted a study on the association between lactylation score and immunocyte infiltration. Our findings revealed a positive correlation between lactylation score and the presence of macrophages, with a specific emphasis on M2-type macrophages (**Figures 5B, C**). Furthermore, the lactylation model showed strong associations with various oncogenic pathways including WNT, VEGF, TOLL-LIKE RECEPTOR, and T-CELL RECEPTOR as revealed by KEGG and Hallmark enrichment analysis (**Figure 5D**). In addition, analysis using Hallmark enrichment showed that the lactylation model was linked to HALLMARK TGF-BETA SIGNALING and HALLMARK WNT BETA CATENIN SIGNALING (**Figure 5E**). The results indicate that a higher lactylation score correlates with elevated proliferation, metastasis, and invasion capabilities in gastric cancer. To gain a deeper understanding of the immunological characteristics across different risk subgroups, we conducted an analysis of genetic mutations. Through this analysis, we identified the top 20 genes with the highest mutation rates in both the high-risk and low-risk subgroups (**Supplementary Figures S2A, B**). Surprisingly, the low-risk group showed a higher mutation rate in these genes. In particular, we discovered that missense mutations were the most common mutation type identified. Among the identified genes, TTN, TP53, and MUC16 had the highest mutation rates, exceeding 25% in both groups. It is intriguing to note that these genes demonstrated a higher mutation rate in the low-risk group. Notably, missense mutations were the most commonly observed type of mutation. Among the identified genes, TTN, TP53, and MUC16 exhibited the highest mutation rates, surpassing 25% in both groups.

### 3.4 Evaluation of immunotherapy response

**Figure 6:**
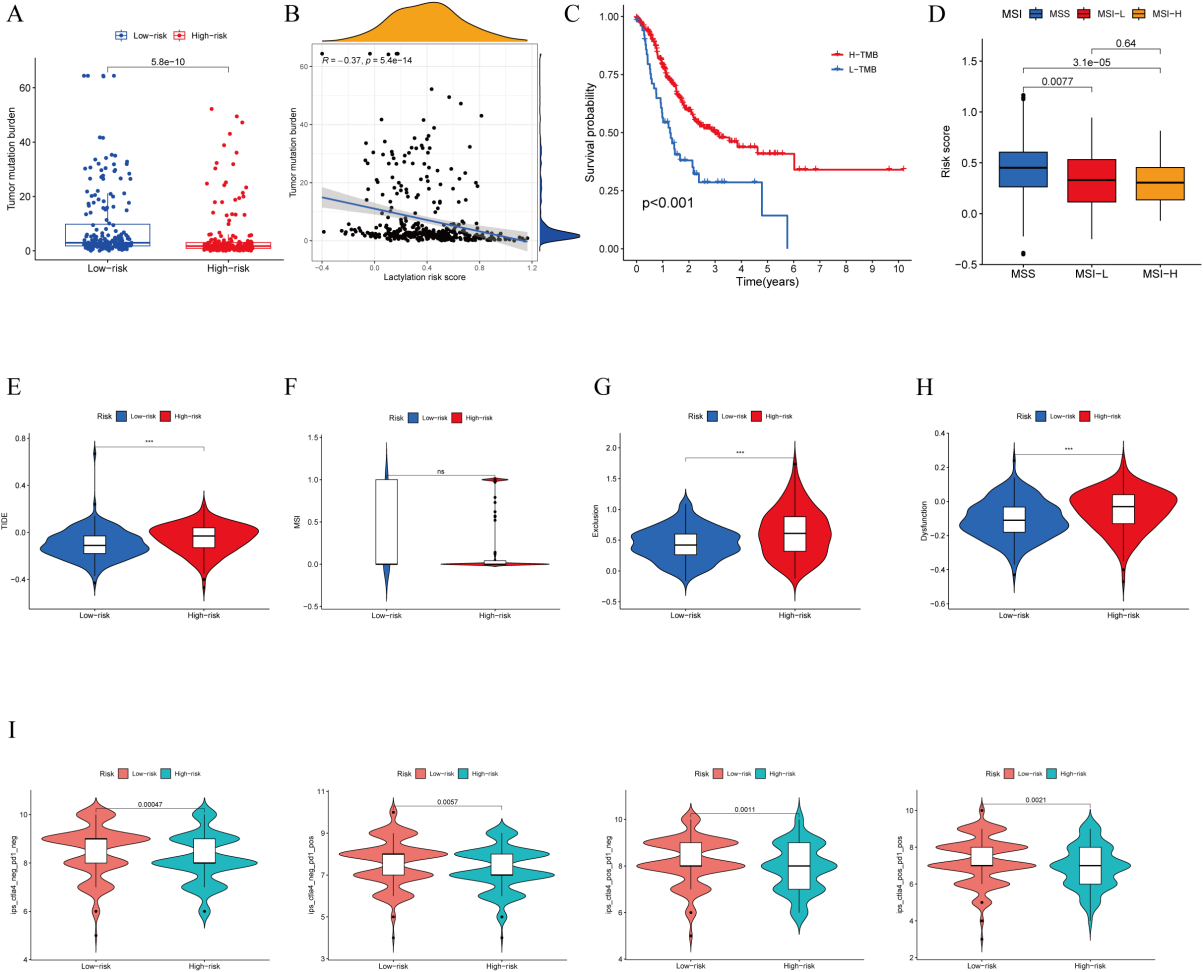
TMB, immune evasion and ICIs. **(A)** TMB score in different lactylation score subgroups and **(B)** the correlation between TMB, high-/low-risk groups **(C)** Kaplan-Meier curve and log-rank test comparethe OS of patients with low or high TMB score. **(D)** Relationship between lactylation score and MSI. **(E–H)** TIDE, MSI, T cell exclusion, and T cell dysfunction, in different lactylation score subgroups,respectively. **(I)** The vioplot of the different expressions of CTLA4 and PD-1 between different lactylation-score groups. ***p<0.001 ns: no significance.

Immunotherapy has shown promising efficacy and minimal serious side effects in the treatment of malignant tumors. It is crucial to understand that immunotherapy may not have the same effectiveness for all patients with cancerous growths. Identifying molecular subtypes is essential for determining which patients will likely see benefits from immunotherapy. During the analysis of patients with high- and low-risk scores, as well as their TMB, significant differences were detected. More specifically, patients in the low-risk score group showcased higher levels of TMB compared to those in the high-risk score group. (**Figure 6A**). Moreover, an inverse relationship was found between the risk score and TMB (**Figure 6B**). In **Figure 6C**, it is shown that the survival rate of the group with high tumor mutational burden (TMB) was notably higher compared to the group with low TMB. The statistical analysis showed a notable variation in the percentage of patients with MSS and MSI-H among various risk score categories. In particular, individuals with microsatellite stable (MSS) tumors exhibited elevated risk scores in contrast to those with microsatellite instability-high (MSI-H) tumors, as shown in (**Figure 6D**). Patients deemed low risk were more likely to benefit from ICI therapy than those deemed high risk. The reason for this finding is that the low-risk subgroup had lower TIDE scores than the high-risk subgroup, as shown in **Figure 6E**. Additionally, our examination uncovered notable distinctions among the two risk categories regarding the T-cell exclusion score (**Figure 6G**) and T cell dysfunction score (**Figure 6H**), with the exception of MSI score (**Figure 6F**). We analyzed the correlation between risk score and IPS in patients with gastric cancer to assess the potential efficacy of clinical immunotherapy in various risk subgroups. The Immune cell Proportion Score (IPS) included immune checkpoints like CTLA-4, PD-1, and PD-L1, which are important markers for forecasting the reaction to ICIs. Utilizing these immune checkpoints, we assessed the potential of ICI treatment in the respective risk subgroups (**Figure 6I**). Surprisingly, a notable rise in immune response was noticed among the low-risk individuals, suggesting that ICIs may provoke a stronger immune reaction in this particular category. Therefore, it can be inferred from these results that individuals in the low-risk category may experience more advantages from immunotherapy as a result of their enhanced immune reaction.

### 3.5 Validation of differentially expressed genes

**Figure 7:**
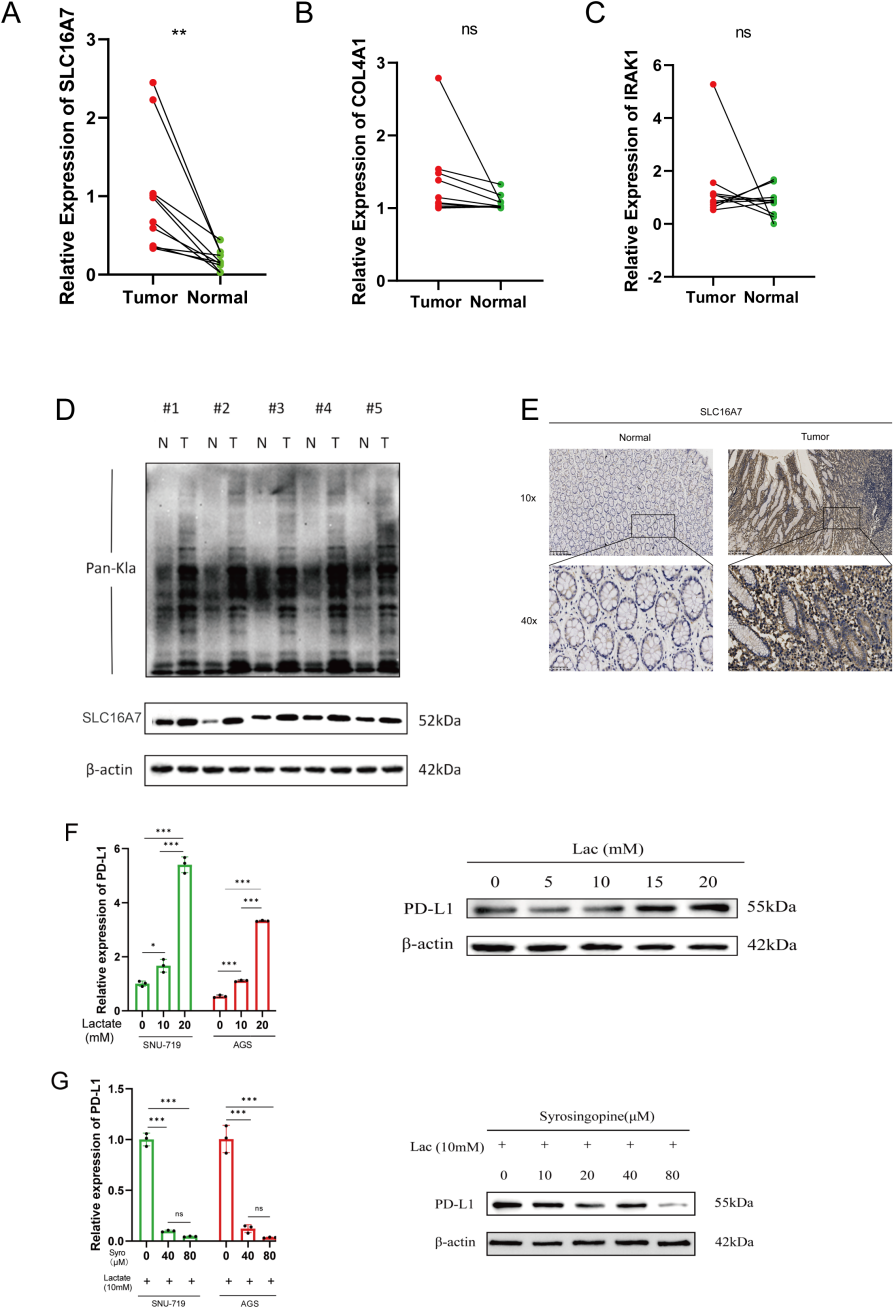
Validation of differentialy expressed genes. **(A–C)** Evaluation of the expression of lactylation-related genes in GC tissues. **(D)** Evaluation of the lactylation levels of paired tumor and normal samples, as well as the expression of SLC16A7 within them. **(E)** SLC16A7 IHC staining in normal and tumor tissues. **(F)** The expression of PD-L1 in gastric cancer cell lines after adding gradient concentration of exogenous lactate acid. **(G)** The expression of PD-L1 in gastric cancer cels after adding 10mM lactate acid and gradient concentration of syrosingopine. *p<0.05;** p<0.01***p<0.001,ns, no significance; Lac, lactate acid; mM, mmol/L; μM, μmol/L.

To validate the expression patterns of the three lactylation-related genes selected for the risk model development, qRT-PCR was employed. In GC tissues, the levels of SLC16A7 expression were found to be increased, while the expression of other genes was not significant according to the results shown in **Figures 7A–C**. As we all know, the gene SLC16A7 encodes a monocarboxylic acid transporter2 (MCT2), which mediates the transport of lactic acid in and out of cells. In order to explore the spread of lactic acid in tumor tissues, we conducted western blot and immunohistochemistry analysis to identify the presence of lactic acid and SLC16A7 in neighboring and tumor tissues. The findings indicated a higher concentration of lactic acid in tumor tissues compared to neighboring tissues. Similarly, the level of SLC16A7 in the tumor tissue is greater than in the normal tissue (**Figures 7D, E**). To assess the influence of lactic acid on PD-L1 expression in gastric cancer cells, we introduced varying concentrations of exogenous lactic acid to the cells. The findings indicated a rise in PD-L1 expression in gastric cancer cells with increasing lactate acid concentration (**Figure 7F**). With the addition of 10 mM lactic acid, the PD-L1 expression decreased as we introduced varying concentrations of syrosingopine (**Figure 7G**).

### 3.6 SLC16A7 reduction impairs T cell killing of tumors

**Figure 8:**
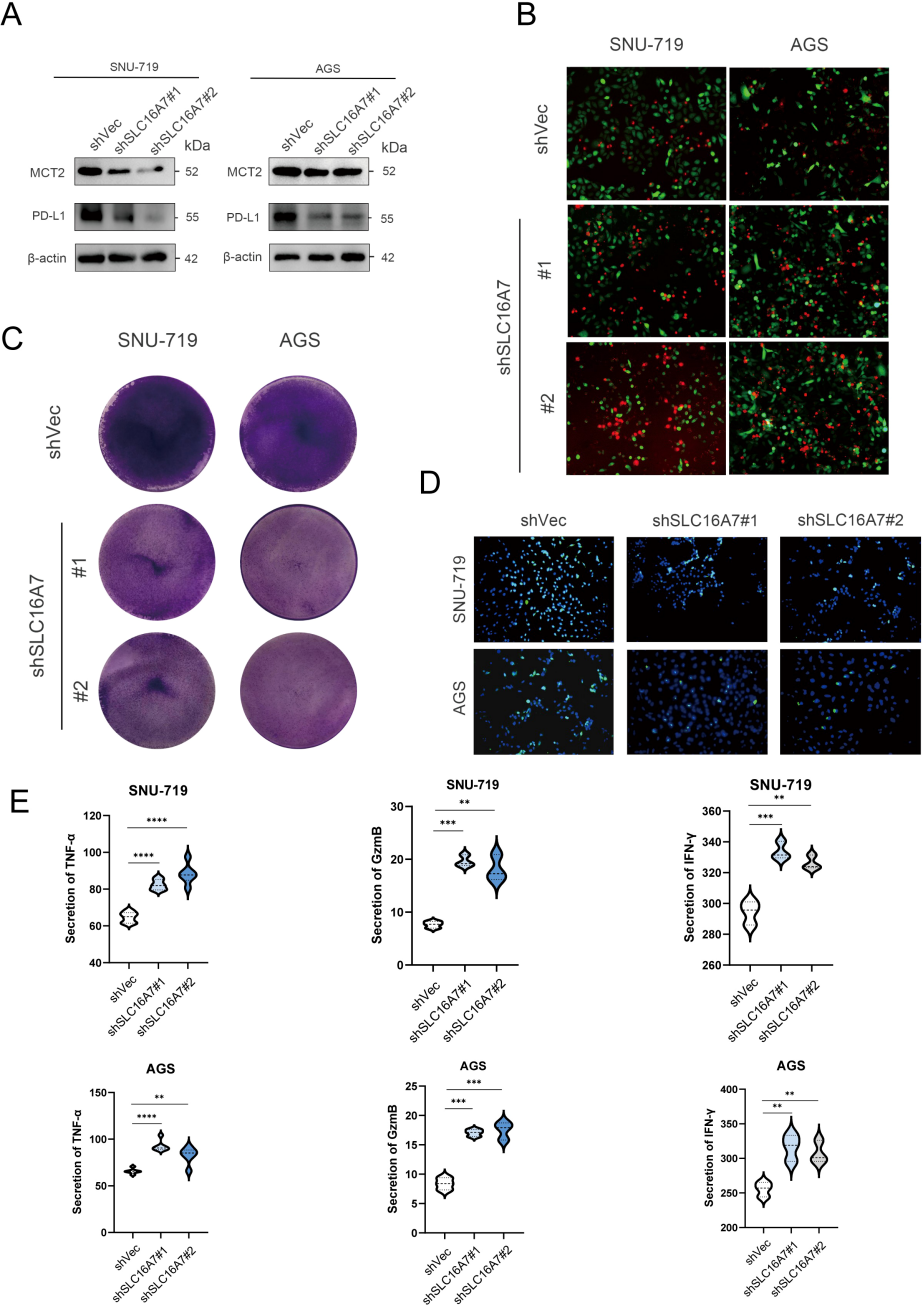
SLC16A7 regulates PD-L1 and *in vitro* cytotoxicity assay. **(A)** Western blot of SLC16A7 knockdown SNU-719 and AGS cells. **(B, C)** T cell-mediated tumor cell killing assay in SLC16A7 knockdown SNU-719 and AGS cells. Representative phase, red fluorescence (dead cells), and green fluorescence (GFP/live cells) merged images are shown. **(D)** Fluorescence microscopy showing the interaction between PD-L1 and PD-1. Representative phase, blue fluorescence (nucleus), and green fluorescence (green fluorescent-labeled PD-1/Fc protein) merged images of SLC16A knockdown in the SNU-719 and AGS cell. **(E)** ELISA analysis on IFN-γ, TNF-α and GzmB of supernatant after co-culture T cells with SNU-719 and AGS cell. **p<0.01; ***p<0.001; **** p<0.0001.

T cells are the main tumor killing cell type in the TME. To further investigate whether SLC16A7 has an effect on T cell killing of tumors, we established stable SLC16A7 knockdown GC cell. Western blot analysis confirmed a significant reduction in SLC16A7 protein levels, indicating successful knockdown. With the knockdown of SLC16A7, the expression of PD-L1 also decreases accordingly (**Figure 8A**). Following SLC16A7 knockdown, we co-cultured SNU-719 and AGS cells with T cells. *In vitro* cytotoxicity assays demonstrated that SLC16A7 depletion significantly decreased T cell-mediated killing (**Figures 8B, C**). PD-L1 and PD-1 interaction assays showed that the level of binding PD-1 fluorescent protein was decreased after SLC16A7 knockdown (**Figure 8D**). In addition, the expressions of inflammatory cytokines including IFN-γ, TNF-α, and GzmB, decreased with SLC16A7 knockdown (**Figure 8E**). This observation indicated that the loss of SLC16A7 in tumor cells influenced the immunosuppressive tumor microenvironment and led to the bad antitumor immune response. These findings demonstrated that SLC16A7 plays an important role in the regulation of TME for the development of antitumor immunity.

### 3.7 Lactic acid promotes the polarization of macrophages to the M2 type

To further study the effects of lactate on immune cells in the tumor microenvironment *in vitro*, we collected the supernatants of tumor cells from the blank control group and the SLC16A7 knockdown group to culture M0 macrophages. The Western blot results showed that, compared to the blank control group, M0 macrophages cultured with the supernatant from the SLC16A7 knockdown group expressed less CD163 (**Supplementary Figure S3A**), indicating a reduced polarization of M0 cells to M2. Immunofluorescence results were consistent with the immunofluorescence findings (**Supplementary Figure S3B**).

## 4 Discussion

Lately, an increasing amount of proof has uncovered the various functions of lactate in the biology of tumors. Lactate not only acts as the main immediate energy supply for tumor cells, but also supports various functions including tumor development, spread, drug resistance, and immune system suppression. These effects are accomplished through diverse mechanisms, encompassing the acidification of the immune microenvironment and the upregulation of proteins that confer resistance to tumors (17). Researchers in the University of Chicago have shown that lactate is a key player in epigenetic modifications, affecting macrophage polarization by histone lactylation (18). Remarkably, histone lysine lactylation, which is influenced by glycolysis, is a widespread modification observed in both human and mouse cells. This process of lactylation is particularly responsive to fluctuations in lactate concentrations, which are affected by the rate of glycolysis or the amount of lactate present. Therefore, the modification of proteins by lactylation is a molecular mechanism that follows glycolysis and emphasizes the important role of lactate in controlling cellular functions.

Two new findings in this investigation have been made. Initially, we developed a gene signature related to lactylation to forecast the outcome of stomach cancer by analyzing genes with varying expressions, such as COL4A1, SLC16A7, and IRAK1. The association between lactylation risk and the immunosuppressive tumor microenvironment (TME), as well as the discovery of predictive biomarkers for immunotherapy response in gastric cancer, was uncovered. To the best of our knowledge, this study is the first to demonstrate the potential of a lactylation-based prognostic risk model in predicting the response to immunotherapy in gastric cancer. Furthermore, we discovered that the inhibition of monocarboxylate transporters (MCTs) for the first time reduced immune evasion in gastric cancer cells by downregulating the expression of PD-L1. The findings suggest that the lactylation risk score, derived from the prognostic model, could potentially function as a predictive biomarker for the immune response in gastric cancer. This could potentially enhance the effectiveness of anti-PD-1 immunotherapy as a promising therapeutic target.

COL4A1, SLC16A7 or IRAK1 are prognostic biomarkers in gastric cancer (19–21). It is challenging, nevertheless, for a single gene to offer patients with gastric cancer strong predictive performance (22). As a result, using a multiple gene model to forecast a cancer patient’s prognosis is becoming more common (23–28). In fact, a number of studies have built predictive models for individuals with osteosarcoma, colon cancer, and breast cancer using various lactylation risk score. Here, we combined patient clinicopathological data with COL4A1, SLC16A7 and IRAK1 to create a predictive nomogram for gastric cancer. While the AUC values of 0.665, 0.637, and 0.662 for 1-year, 3-year, and 5-year predictions may not represent optimal performance, they still indicate moderate prognostic value. There are several potential reasons for the model’s relatively lower performance. Firstly, lactate metabolism is a complex and multifactorial process, influenced by a wide range of variables beyond the genes included in our model, such as environmental factors, additional molecular pathways, and patient-specific clinical variables. This complexity could reduce the model’s discriminatory power. Additionally, the heterogeneity of cancer types and stages within the TCGA dataset might also have contributed to the variability in prediction accuracy across different time points. The model may perform differently depending on the specific clinical context of the patients, such as tumor type, stage, and treatment history, which were not explicitly accounted for in our analysis. In comparison with other published models, it is worth noting that many of those models include a broader range of clinical and molecular data, such as imaging, multi-omics integration, or patient demographic information, which can significantly improve predictive accuracy. In contrast, our model focused solely on lactate metabolism-related genes. However, despite these limitations, our model still demonstrates a level of prognostic capability that warrants further exploration, especially when integrated with additional data types to enhance its predictive performance.

With the continuous advancements in sequencing and spectrometry technologies, MCT protein family are being constantly discovered and identified (29). There are currently 14 known proteins belonging to the SLC16 gene family (30). Based on the properties of the protein transport substrates, we were able to classify proteins from the SLC16 gene family into 3 classes. One type of MCTs is characterized by the fact that MCTs require proton involvement when transporting substrates, and we can also call this type of MCT proton-coupled monocarboxylic acid transporter (31). The main types of MCTs are MCT1 (SLC16A1), MCT2 (SLC16A7), MCT3 (SLC16A8), and MCT4 (SLC16A3). Recent research indicates that SLC16A7is present in various cancer cells, with varying levels of expression in normal tissues and cancer cells, suggesting its potential as a tumor biomarker (31, 32). The study discovered that the localization of SLC16A7 to peroxisomes is linked to the malignant transformation of prostate cancer (33), as determined by assessing intracellular SLC16A7 expression. SLC16A7 is not limited to prostate cancer, it has also been detected in various tumor cells, including those of lung and colon cancer.

High levels of MSI have been found to be closely associated with improved prognosis in gastric cancer patients who receive immunotherapy. MSI is a biomarker that indicates a defect in the DNA mismatch repair system, leading to an increased number of genetic mutations. These mutations result in the production of neo-antigens, which are unique proteins that can be recognized by T cells. A higher TMB, which refers to the total number of mutations in a tumor’s DNA, can also lead to the production of more neo-antigens. This increased antigenicity enhances the chances for T cell recognition and activation, ultimately improving the outcomes of ICI therapies (34). We have demonstrated a negative correlation between lactylation risk and TMB/MSI in gastric cancer patients. It suggests that patients with a low lactylation risk are more likely to benefit from immunotherapy. This finding is consistent with the existing literature, which suggests that a favorable tumor microenvironment, characterized by lower lactylation risk and higher TMB/MSI, is associated with better responses to immunotherapy. Lower lactylation risk indicates reduced lactate production by tumor cells, which can contribute to an immunosuppressive environment. In contrast, higher TMB and MSI indicate a higher mutational load, leading to the production of neo-antigens that can activate the immune system and enhance response to immunotherapy. Therefore, patients with low lactylation risk and high TMB/MSI may have a more immunogenic tumor profile, making them more responsive to immunotherapy. This finding aligns with existing literature that suggests patients with higher TMB and MSI tend to have better outcomes with immunotherapy (35). Previous studies have indeed shown that TTN mutations are associated with high immunogenicity and an inflammatory tumor immune microenvironment in lung adenocarcinoma. These mutations can lead to the production of neo-antigens, which can trigger an immune response and enhance the efficacy of ICIs. Consequently, patients with TTN mutations may exhibit a more favorable objective response and improved survival when treated with ICIs (36, 37). The TIDE score, which reflects the ability of tumor cells to evade immune surveillance, was found to be higher in the high lactylation risk group compared to the low lactylation risk group. This suggests that lactylation of TME may play a role in promoting immune evasion in gastric cancer. We also found that gastric cancer tissues from patients with a high lactylation risk showed increased infiltration of macrophages 2. The study found a positive correlation between lactate concentration and PD-L1 expression as well as the level of lactylation in human gastric cancer tissues. PD-L1 is a protein that is often overexpressed in cancer cells and interacts with PD-1 receptors on immune cells, leading to immune suppression. The positive correlation suggests that lactylation may contribute to the upregulation of PD-L1, potentially leading to immune dysfunction in gastric cancer. Treated with syrosingopine, the PD-L1 expression decreased as the concentration of syrosingopine elevated. Based on the strong associations observed between lactylation risk, PD-L1 expression, TMB, and MSI, it is reasonable to conclude that lactylation risk, including SLC16A7, may have potential as a biomarker for predicting the response to immunotherapy in gastric cancer.

MCT4 positively regulates the expression of PD-L1 in breast cancer cells by releasing lactate, and it stabilizes PD-L1 by promoting its glycosylation through the classical WNT pathway (38). In addition, lactylation, a newly discovered post-translational modification in recent years, has attracted considerable attention. Lactylation has been found in different pathophysiological states and leads to various biological effects, though only a few mechanisms have been elucidated. Lactic acid exerts its PD-L1 induction effect by lowering cAMP levels and activating TAZ (39). Yu et al (40) discovered that histone lactylation is associated with poor prognosis in ocular melanoma, and further demonstrated that the mechanism of histone lactylation affects transcription factor recognition of TP53 modifications and mediates its degradation, promoting the occurrence of ocular melanoma.

When lactic acid from tumor cells is transported to the tumor microenvironment through the lactate transporter SLC16A7, it can induce the conversion of M0 macrophages to the M2 type, suppressing the body’s immune function. This process is inhibited when the SLC16A7 gene is knocked out. We conducted a literature search to explore how lactic acid secretion might inhibit the functions of immune cells in the immune microenvironment, which aligns with our research findings (41–43).

Despite the strengths of our study, it is essential to acknowledge its limitations. Further research, considering larger sample sizes, diverse patient populations, and mechanistic studies, will help validate and expand upon these findings, ultimately leading to a more comprehensive understanding of the influence of lactylation in GC. Validation of the lactylation score’s efficacy in predicting reaction to ICI therapy requires extensive clinical trials.

## 5 Conclusion

To summarize, the lactylation score has the potential to contribute to the molecular classification of GC by identifying distinct immune infiltration patterns and genomic instability profiles. Moreover, it could serve as a valuable tool for assessing patient response to ICI treatment.

## Data Availability

The original contributions presented in the study are included in the article/**Supplementary Material**. Further inquiries can be directed to the corresponding authors.

## Supplementary material

Supplementary Figure 1 Kaplan-Meier survival curves of three lactylation genes. **(A, B)** GC patients with high expressionof COL4A1 and SLC16A7 had good prognosis. **(C)** GC patients with high expression of IRAK1 had poor prognosis.

Supplementary Figure 2 Significantly muted genes in GC samples of the high-and low-risk groups.

Supplementary Figure 3 Lactate acid promotes M2 polarization of M0 macrophages. **(A)** Western blot analysis of CD163 expression in M0 macrophages co-cultured with supernatant of gastric cancer cells. **(B)** Immunofluorescence detection of CD163 expression in M0 macrophages co-cultured with supernatant of gastric cancer cells.
