# Supplementary figures and images for: Lactylation-related gene signature accurately predicts prognosis and immunotherapy response in gastric cancer
Source: Front Oncol. 2024 Nov 28;14:1485580. doi: 10.3389/fonc.2024.1485580 (PMC11634757; doi:10.3389/fonc.2024.1485580)

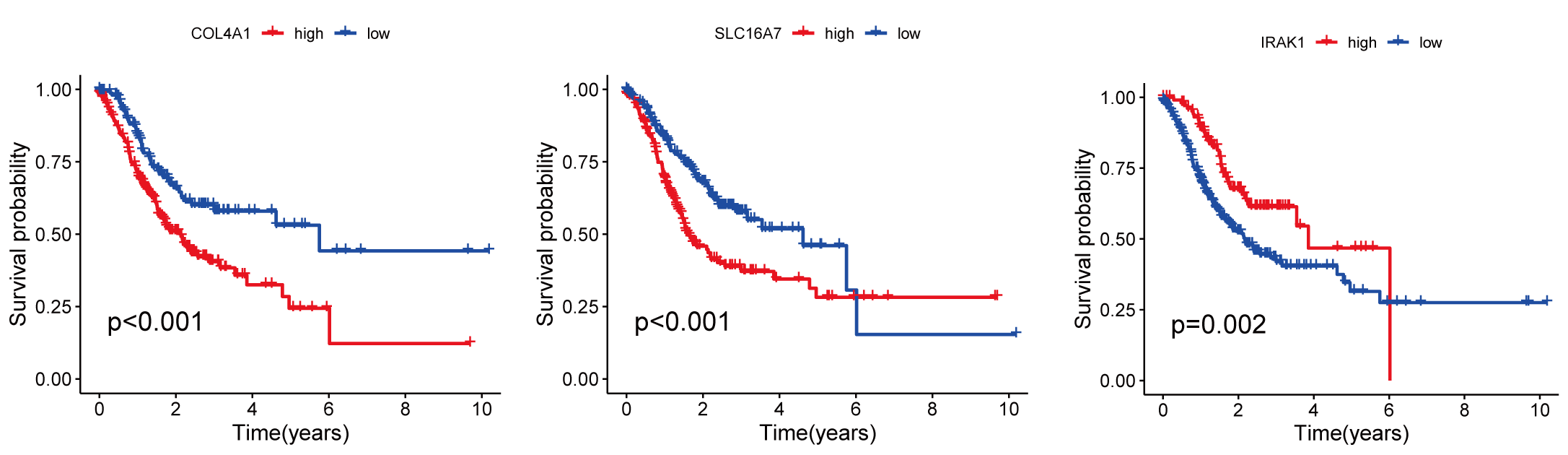

Supplement: Supplementary Figure 1 — Kaplan-Meier survival curves of three lactylation genes. (A, B) GC patients with high expressionof COL4A1 and SLC16A7 had good prognosis. (C) GC patients with high expression of IRAK1 had poor prognosis. [file Image1.tif]

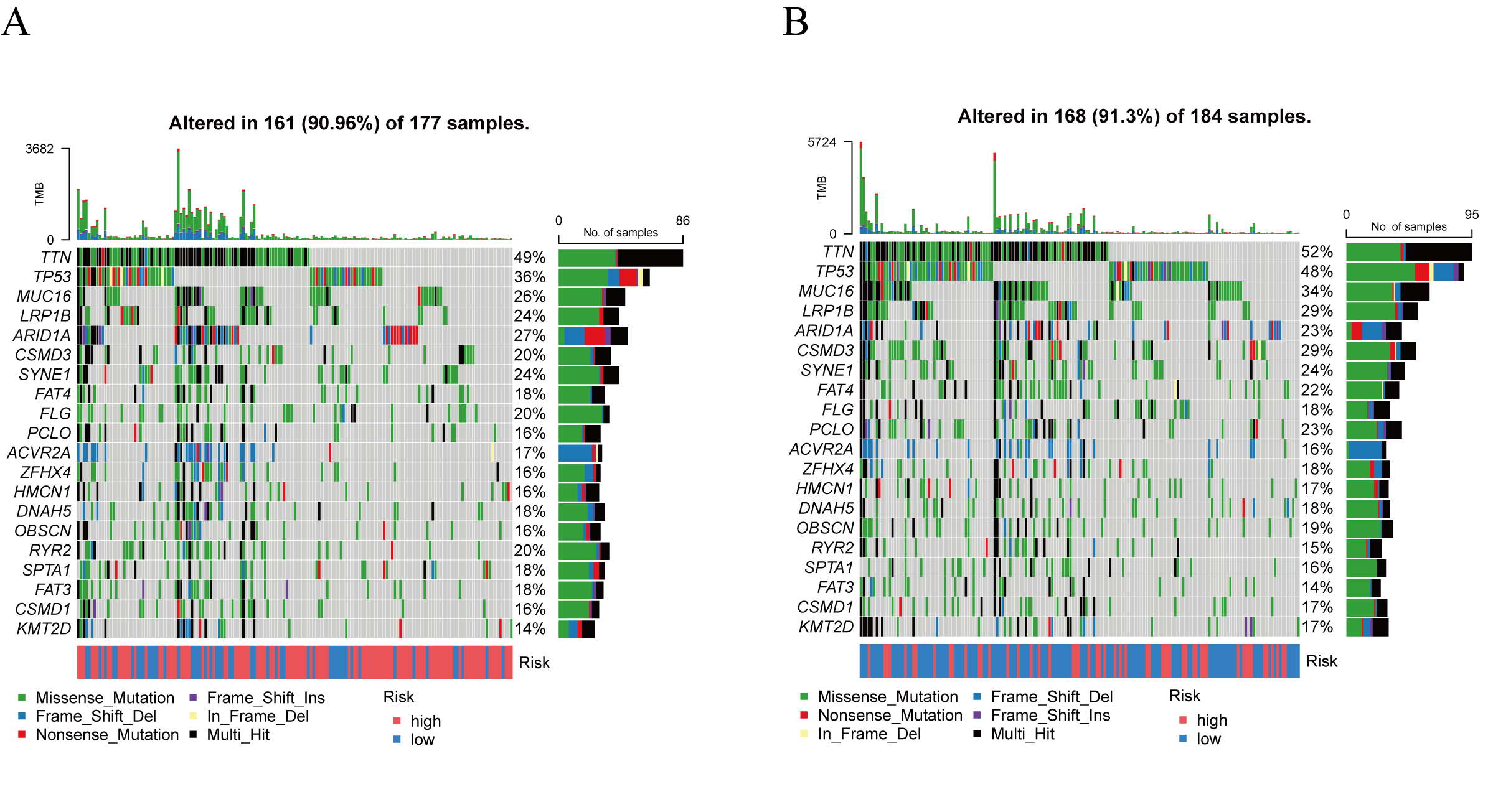

Supplement: Supplementary Figure 2 — Significantly muted genes in GC samples of the high-and low-risk groups. [file Image2.tif]

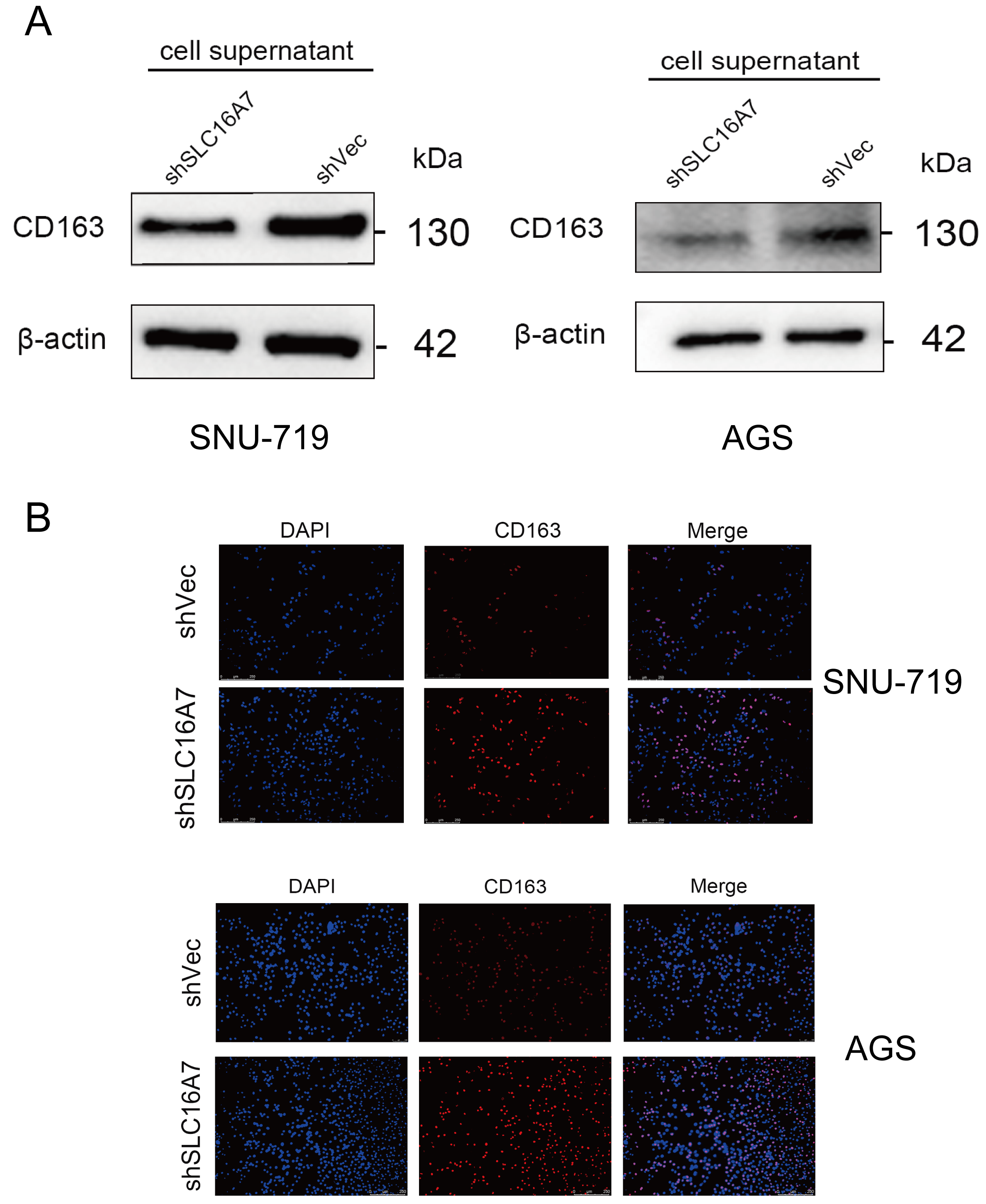

Supplement: Supplementary Figure 3 — Lactate acid promotes M2 polarization of M0 macrophages. (A) Western blot analysis of CD163 expression in M0 macrophages co-cultured with supernatant of gastric cancer cells. (B) Immunofluorescence detection of CD163 expression in M0 macrophages co-cultured with supernatant of gastric cancer cells. [file Image3.tif]
